# Visualization of poikilothermia using handheld thermography

**DOI:** 10.1002/ccr3.1088

**Published:** 2017-07-20

**Authors:** Yohei Okada, Hiromichi Narumiya

**Affiliations:** ^1^ Department of Emergency and Critical Care Medicine Japanese Red Cross Society Kyoto Daini Red Cross Hospital 355‐5 Haruobicho Kamigyoku Kyoto 602‐8026 Japan

**Keywords:** Forward looking infrared, thermal imaging, veno‐arterial extracorporeal membrane oxygenation (ECMO)

## Abstract

Poikilothermia is a fundamental symptom of acute limb ischemia (ALI) and is known as “6Ps”. Herein, we present the visualization of poikilothermia caused by ALI using handheld thermography. We believe that handheld thermography could be useful to assess poikilothermia objectively as a supplementary method to physical examination.

## Case Presentation

**Figure 1:**
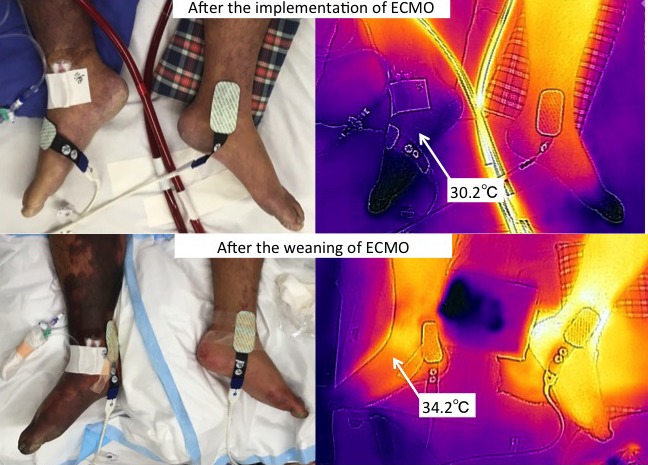
Poikilothermia detected using handheld thermography.

Poikilothermia, also known as “6Ps”, is a fundamental symptom of acute limb ischemia (ALI) 1; however, this condition cannot be assessed objectively. Herein, we present a case of a 27‐year‐old man whose right foot was diagnosed with ALI due to insertion of a cannula into the right femoral artery for veno‐arterial extracorporeal membrane oxygenation (ECMO). The condition had improved by day 2 following weaning from ECMO. These images represent poikilothermia objectively using handheld thermography (FLIR‐One for iOS) (Fig. 1).

We suggest that handheld thermography could be useful to assess poikilothermia objectively as a supplementary method to physical examination.

## Authorship

YO: has a responsibility for the patient care and wrote the manuscript. HN: supervised it.

## Conflict of Interest

None.
